# Inferring dynamic information from protein structures by Gaussian integrals and deep learning

**DOI:** 10.1093/bioinformatics/btag446

**Published:** 2026-06-24

**Authors:** Felipe Vilicich, Nicolás Bottino, Zhaoqian Su, Shanye Yin, Yinghao Wu

**Affiliations:** Department of Systems and Computational Biology, Albert Einstein College of Medicine, Bronx, NY 10461, United States; Departamento de Ciencias Aplicadas, Universidad Siglo 21, Córdoba X5147, Argentina; Data Science Institute, Vanderbilt University, Nashville, TN 37212, United States; Department of Pathology, Albert Einstein College of Medicine, Bronx, NY 10461, United States; Department of Systems and Computational Biology, Albert Einstein College of Medicine, Bronx, NY 10461, United States

## Abstract

**Motivation:**

Protein dynamics are central to function, but experiments and molecular dynamics (MD) simulations remain costly, low-throughput, and difficult to compare across protocols. Scalable structure-based methods are needed to infer dynamics from static protein structures.

**Results:**

We present a deep learning framework that predicts protein dynamics from 30-dimensional Gaussian integral (GI) descriptors of Cα backbone topology. Using 1374 ATLAS protein chains with MD-derived RMSF, GI stratified proteins into fold-relevant clusters enriched for secondary structure, sequence homology, and ECOD families. An attention-based 1D-CNN classified flexible versus non-flexible proteins with test AUC = 0.772 and separated slow-mode– from fast-mode–dominated dynamics with AUC = 0.91. Regression models recovered mean RMSF (Pearson *r = *0.72; *R*² = 0.46) and slow-mode RMSF more accurately (Pearson *r = *0.83; *R*² = 0.62), supporting rapid inference of flexibility and collective-motion bias.

**Availability and implementation:**

Code and data are available on GitHub at: https://github.com/fvilicich/gaussian_integral/blob/main/gaussian_integral_classification.ipynb.

## 1 Introduction

Proteins are essential components in most biological processes in living organisms (Morris *et al*. 2022). The majority of these biomolecules adopt specific three-dimensional structures, which are determined by their primary sequences (Anfinsen 1972). However, protein structures are not static under physiological conditions and undergo continuous conformational changes (Stollar and Smith 2020). This dynamic behavior allows proteins to carry out their functions in the cellular environment (Schmid and Hugel 2020). Conformational variations enable proteins to switch between different functional states, allowing them to respond dynamically to external signals and enhancing their affinities for substrates or interactions with other molecules. These capabilities are fundamental to processes such as enzyme regulation and signal transduction. Therefore, exploring protein dynamics and how they are influenced by protein structure is crucial for gaining deeper insights into their roles in biology.

Information on protein dynamics can be characterized experimentally by methods such as nuclear magnetic resonance (Kleckner and Foster 2011) and fluorescence resonance energy transfer (Yengo and Berger 2010). Nevertheless, the spatial-temporal resolutions of the dynamic information obtained by these methods are limited, preventing them from providing mechanistic insights of protein functions at atomic detail. Moreover, these experimental approaches are time-consuming and labor-intensive, making them unsuitable for large-scale studies or high-throughput screening. Finally, different experimental techniques require proteins to be studied under varying conditions, which may not accurately replicate the physiological environment in which these proteins function in living organisms. This discrepancy poses a challenge in interpreting experimental results and comparing outputs across different techniques.

Computational methods serve as an effective alternative for testing conditions that are currently inaccessible in the laboratory. Among a large variety of computational tools available, molecular dynamics (MD) simulation is specifically designed to study the physical movements and interactions of biomolecules over time (Craig *et al*. 2004, Gottschalk and Kessler 2004, Cailliez and Lavery 2005, 2006, Puklin-Faucher *et al*. 2006, Sotomayor and Schulten 2008, Wan *et al*. 2008, Maruthamuthu *et al*. 2009). By solving Newton’s equations of motion for each particle in a system, this method simulates molecular dynamics at the atomic level. MD simulations have been widely applied to evaluate the effects of conformational changes on enzyme catalysis, allosteric pathways, ligand-receptor recognition, and protein complex assembly (Hollingsworth and Dror 2018). Despite their versatility, MD simulations require significant computational resources. Systematic testing to simulate large numbers of biomolecules over long time scales is impractical without access to high-performance computing facilities, such as Anton (Shaw *et al*. 2021). Furthermore, variations in software, force fields, system settings, or simulation protocols can produce inconsistent results (Serafeim *et al*. 2016), even for the same proteins, making it extremely difficult to compare results from different research groups. As a result, there is a growing demand for computationally efficient tools that can systematically analyze protein conformational dynamics. Such tools should complement MD simulations and provide significant value to the scientific community by advancing the understanding of protein function.

Machine learning (ML) offers a promising route toward scalable characterization of protein dynamics. Driven by the rapid growth of structural data in the Protein Data Bank (Laskowski 2016), deep learning has achieved major success in structural biology (Kumar and Srivastava 2024) exemplified by AlphaFold (Baek *et al*. 2021, Abramson *et al*. 2024) and RoseTTAFold (Humphreys *et al*. 2021). More recently, MD simulations have been used to train ML models that generate physically realistic conformational ensembles (Elia Venanzi *et al*. 2024), while generative approaches (e.g. variational autoencoders and adversarial networks) helped us learn compact representations of conformational landscapes that support sampling of novel structures (Tian *et al*. 2021, Zheng *et al*. 2023). Reinforcement learning has also been integrated with MD trajectories and free-energy maps to accelerate exploration of conformational space (Kolossváry 2026). In parallel, several studies have explored scalable prediction of protein flexibility directly from sequence. Methods such as PEGASUS (Vander Meersche *et al*. 2025) and Flexpert (Kouba *et al*. 2025) leverage evolutionary information to infer MD-derived flexibility at high throughput. Complementing these approaches, novel structure-based methods such as Backflip (Viliuga *et al.* 2025) predict protein dynamics directly from three-dimensional coordinates, bypassing sequence-derived features. Together, these advances reflect a shift from static structure prediction toward modeling protein dynamics across multiple input modalities.

In this paper, we present a deep learning framework to infer dynamic information from protein structures. By leveraging a recently developed database, ATLAS (Vander Meersche *et al*. 2024), we can access the all-atom MD simulation results for more than one thousand proteins with diverse tertiary structures. We further transferred these protein structures into high-dimensional vectors by Gaussian integrals (GIs)) (Rogen and Fain 2003). GIs provide a simplified representation of protein three-dimensional folds, in which the Cα backbone trace is modeled as an oriented open spatial curve and a series of generalized Gauss integrals are computed from this curve. The two lowest-order integrals, writhe and average crossing number, serve as natural measures of backbone entanglement. Higher-order Gauss integrals characterize more complex entanglement motifs along the chain. In this manner, a protein’s Cα trace can be compactly represented as a vector of Gauss integral descriptors. The vectors were fed to a 1D convolutional network with an attention layer to predict the amplitudes of protein conformational fluctuations. Based on the cross-validation against the ATLAS database, we showed that our deep learning model can classify proteins as flexible or non-flexible with more than 70% accuracy and can additionally distinguish slow-mode–dominated from fast-mode–dominated dynamics with high discriminative performance. Using regression outputs, we reproduced the average per-protein root-mean-square-fluctuations (RMSF) values derived from MD simulations and predicted slow-mode RMSF with even stronger agreement. Because GIs encode global features of protein shape, these results demonstrate that key aspects of protein dynamics can be decoded from structure without explicit sequence information. In summary, this computationally efficient framework complements atomistic MD and provides a scalable approach for high-throughput inference of protein flexibility and collective-motion biases.

## 2 Methods

### 2.1. Dataset

**Figure 1:**
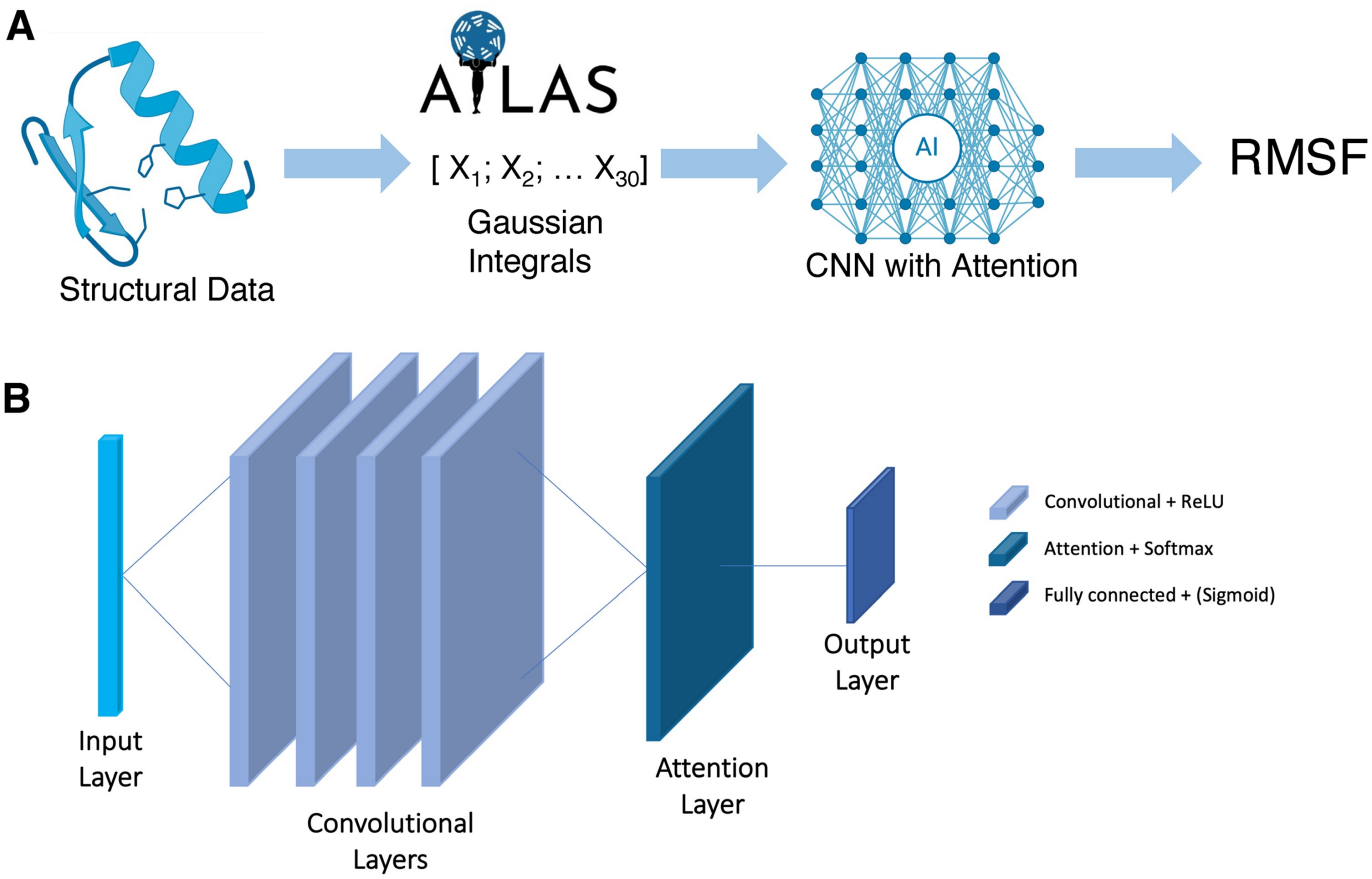
Outline of the problem. (A) Flow chart showing our approach to solve the problem. (B) Architecture of the model for regression and classification tasks. Protein structure and neural network icons were created in BioRender. Vilicich, F. (2026) https://BioRender.com/2jd09y0.

All data used in this study were drawn from the ATLAS database of standardized all-atom molecular dynamics (MD) simulations (Vander Meersche *et al*. 2024). ATLAS comprises trajectories for 1,374 protein chains representing 1,149 non‐redundant ECOD X-class domains, each simulated in triplicate for 100 ns using GROMACS with the CHARMM36m force field. From these trajectories, per‐residue RMSF of Cα atoms were computed and averaged across replicates to yield a single RMSF value per protein, which serves as our target variable (Fig. 1A).

### 2.2. Gaussian integrals

To transform 3D structures into fixed-length feature vectors, we computed 30 topological invariants, also known as generalized GIs, capturing global curve properties of the protein backbone (writhe, average crossing number, and higher-order correlations) (Rogen and Fain 2003). These GI descriptors, normalized to unit variance across the dataset, serve as input features to our convolutional network with attention (Fig. 1A).

### 2.3. GNM normal mode analysis of protein flexibility

Protein flexibility was estimated using Gaussian Network Model (GNM) normal mode analysis on Cα-only representations, with residue–residue contacts defined by a 10 Å cutoff. We computed up to 100 non-trivial modes and separated the spectrum into low-frequency “slow/soft” global modes (first 20%) and high-frequency “fast/stiff” local modes (last 20%), as in established GNM interpretations (Li *et al*. 2016). Residue-wise RMSF was computed from the selected eigenmodes and summarized per protein as the mean RMSF across residues.

### 2.4. ANM-based visualization of representative protein motions

To qualitatively illustrate the types of motion captured by the slow-fraction metric, we performed anisotropic network model (ANM) analysis on selected representative proteins using ProDy. ANM was constructed using Cα-only representations with a 15 Å cutoff to define residue–residue interactions. Low-frequency normal modes were computed, and motions were visualized by perturbing the structure along individual modes using 100 interpolation steps and a fixed amplitude corresponding to 4 Å RMSD. Resulting conformational trajectories were exported as multi-model PDB files and visualized in VMD to generate vector field representations and supplementary animations (Supplementary Movie 1, available as supplementary data at *Bioinformatics* online).

### 2.5. Deep learning models

Our classifier is a one-dimensional convolutional neural network (1D-CNN) with an embedded attention mechanism (Hernández and Amigó 2021) applied to the GI sequence. Specifically, the model treats each GI vector as a single-channel time series of length 30, resulting in input tensors of shape (batch size × 1 × 30). The network consists of four stacked 1D convolutional blocks (kernel size = 3, padding = 1, no dilation), each followed by a ReLU activation, enabling the learning of hierarchical patterns across the sequence. An attention layer is then applied to compute learned importance weights over the 30 positions, aggregating them into a single 128-dimensional feature vector. This vector is passed through a fully connected layer that maps it to a single logit, which is then transformed via a sigmoid activation to produce a probability score (Fig. 1B). The attention mechanism was included to allow the model to learn which positions along the GI sequence are most informative for classification. Unlike traditional pooling methods that treat all positions equally, attention dynamically weights each of the 30 input positions based on their contribution to the final prediction. This not only improves performance by focusing the model’s capacity on relevant features, but also enhances interpretability by enabling us to probe which regions of the structural encoding influence the classifier’s decision.

### 2.6. Training and validation

Five-fold cross-validation was performed on the 1099 proteins (with a randomly held-out test set of 275). In each fold, 80% of the 1099 served for training and 20% for validation, stratified by class (flexible and non-flexible). We used BCELoss, Adam (learning rate = 10^-^³, weight_decay = 10^−4^), batch size = 32, and early stopping (patience = 7, max epochs = 50). After identifying the fold with the best validation accuracy, we retrained that model on all 1099 examples and evaluated its final performance on the independent 275-protein test set. Loss curves for training and validation can be found in Supplementary Materials.

### 2.7. Targeted ablation studies

To localize which GI positions contribute most to model predictions, we performed targeted feature ablation and attribution analyses across all tasks in this study. For each GI position, we estimated importance using four complementary approaches and summarized results as heatmaps of normalized ranks across GI positions (Figs. S1C–D and S2C–D, available as supplementary data at *Bioinformatics* online). Within each approach, GI positions were ranked from most to least important and rescaled to [0,1] to enable direct comparison across methods with different units and scales. Univariate associations: We computed Spearman rank correlations between each GI position and the task target using the training data, ranking positions by ∣ρ|. Single-feature baselines: For each GI position, we trained a univariate baseline model using that single feature and ranked positions by held-out performance (using the task-appropriate metric). Permutation importance (1D-CNN): Using the trained attention-based 1D-CNN, we permuted one GI position across samples while keeping all other positions fixed, and quantified importance as the resulting drop in performance relative to the unpermuted baseline, averaged across permutations. Attention-based contrasts: We summarized the model’s attention weights over GI positions and quantified per-position differences between output groups, ranking positions by the magnitude of the attention difference.

## 3 Results

### 3.1. GI-based structural stratification of the ATLAS database

**Figure 2:**
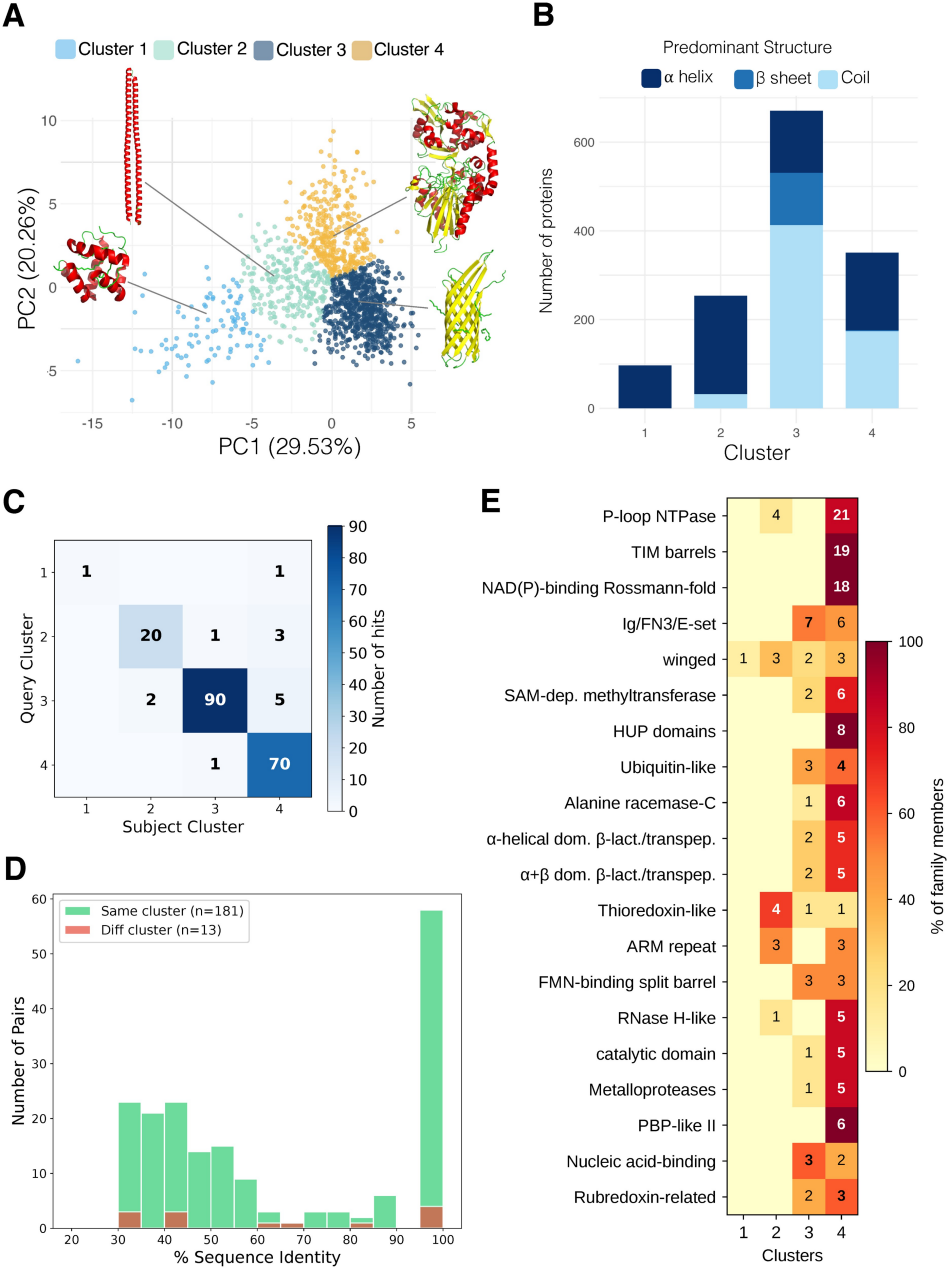
GI-based structural stratification of the ATLAS database. (A) PCA of 30-dimensional GI descriptors for 1374 ATLAS proteins with examples. (B) Predominant secondary structure composition within each GI cluster, where each protein is assigned to α-helix, β-sheet, or coil by a majority rule using ATLAS residue-level annotations. (C) Cluster-to-cluster mapping of homologous protein pairs identified by all-vs-all BLAST (*n = *194 pairs), visualized as counts of query/subject cluster assignments. (D) Sequence identity distribution for homologous pairs stratified by whether both proteins fall within the same GI cluster versus different clusters. (E) ECOD X-class family enrichment across GI clusters, shown as the percentage of family members assigned to each cluster for the most represented families.

We first performed a principal component analysis (PCA) (Gewers *et al*. 2022) of the GIs calculated for 1,374 protein chains from the ATLAS database, revealing four distinct clusters (labeled 1 through 4). The first two principal components (PC1 and PC2) capture 29.53% and 20.26% of the total variance in the dataset, respectively, indicating that nearly half of the structural variability encoded by the GIs can be visualized in this two-dimensional projection (Fig. 2A).

Then, we examined the structural composition of each cluster in terms of secondary structure content. The annotations for α-helix, β-sheet, and coil were obtained directly from the ATLAS database, which provides residue-level assignments for each protein chain. To assign a single predominant structure to each protein, we applied a simple majority rule: the secondary structure type with the highest residue count along the chain was considered the dominant one. For example, a protein composed of 70% α-helix, 20% β-sheet, and 10% coil was categorized as α-helical. Such classification scheme, while reductive, enables a tractable comparison of structural trends across clusters and reveals distinct secondary structure preferences associated with each group. This analysis showed that clusters 1 and 2 are enriched in α-helical proteins, while cluster 3 is dominated by coil-rich proteins, with additional representation from β-sheets and α-helices. Cluster 4 displays a near-even split between α-helical and coil-dominant proteins (Fig. 2B).

All-vs-all BLAST analysis identified 194 homologous pairs among the 1374 ATLAS chains, and the vast majority (181/194; 93.3%) mapped to the same GI-defined PCA cluster (Fig. 2C). Homologous pairs assigned to the same GI cluster spanned a wide range of sequence identities, including many low-to-moderate identity relationships, while cross-cluster homologs were rare (*n = *13) (Fig. 2D). Mapping ECOD X-class families onto the GI clusters revealed distinct family enrichments, with multiple fold families preferentially concentrated within specific clusters (Fig. 2E). Together, these analyses indicate that GI-based clustering recapitulates both sequence homology and fold-family organization, supporting that the GI representation captures meaningful, conserved structural signatures in ATLAS.

### 3.2. Deep learning on GI descriptors classifies flexibility and mode dominance

We next tested whether GI descriptors are predictive of protein dynamics by training a 1D-CNN with attention on 1099 proteins (5-fold cross-validation) and evaluating all reported results on an independent 275-protein test set. We formulated two binary tasks: (i) flexible vs non-flexible, defined by whether the mean per-protein RMSF (averaged over residues) falls above or below the dataset-wide mean, and (ii) slow-mode vs fast-mode dominated, defined by a slow-fraction metric computed as:


$$\mathrm{Slow}\;\mathrm{Fraction}=\frac{\mathrm{RMS}F_{\mathrm{slow}}}{\mathrm{RMS}F_{\mathrm{slow}}+\mathrm{RMS}F_{\mathrm{fast}}}$$

Where RMSF_slow_ and RMSF_fast_ are the per-protein RMSF contributions from low and high frequency GNM mode subsets, respectively; proteins were labeled slow-dominated if their Slow Fraction was above the dataset-wide median and fast-enriched otherwise.

**Figure 3:**
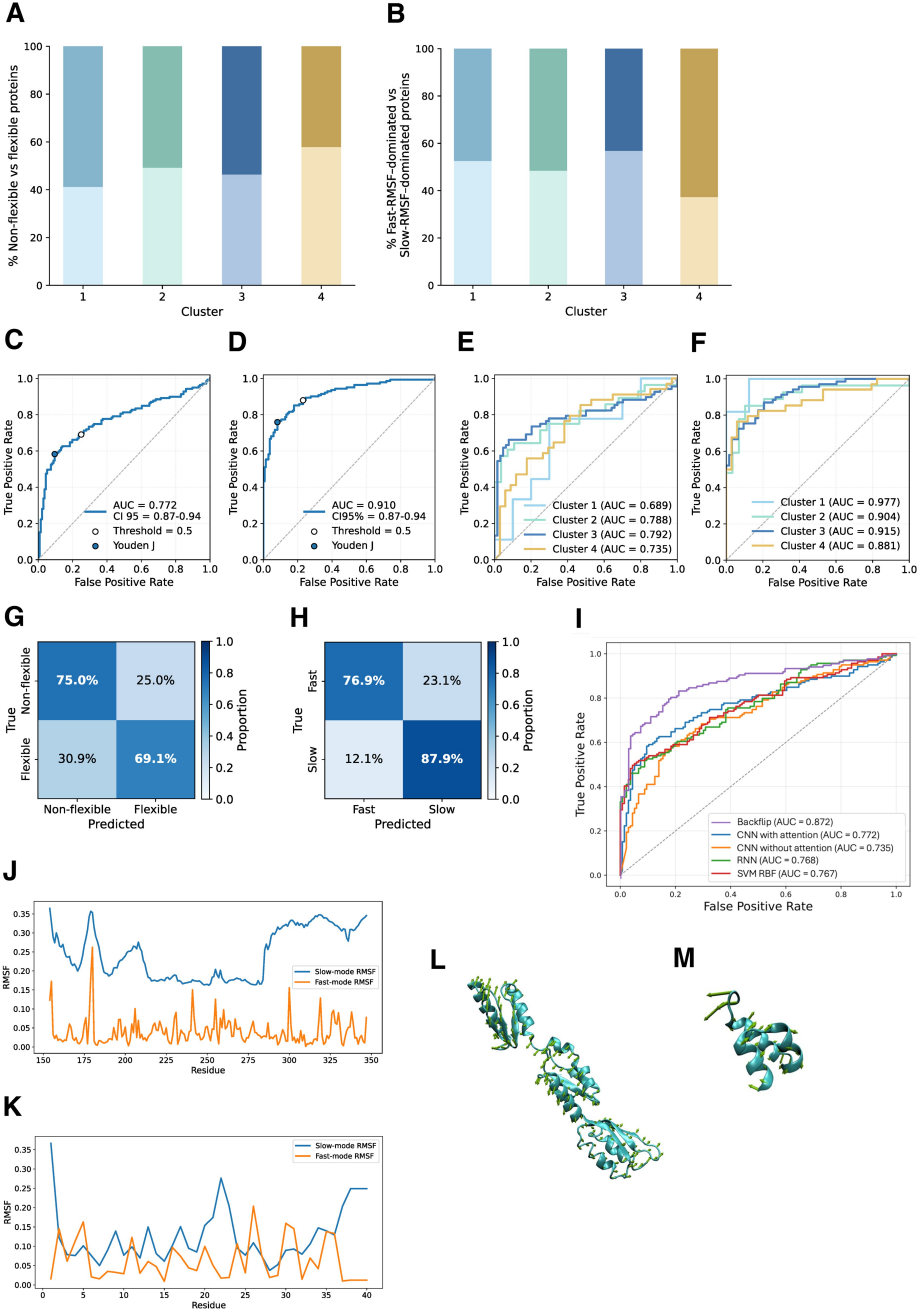
Deep learning on GI descriptors classifies flexibility and mode dominance. (A) Distribution of flexible versus non-flexible proteins across GI clusters in the test set where flexibility is defined by mean per-protein RMSF relative to the dataset-wide mean. (B) Distribution of slow-mode–dominated versus fast-mode–dominated proteins across GI clusters in the test set where dominance is defined by the slow-fraction metric thresholded at the dataset-wide median. (C) ROC curve for flexible vs non-flexible classification on the test set using the 1D-CNN with attention; the optimal threshold is marked using the Youden J statistic, and AUC with 95% CI is reported. (D) ROC curve for slow-mode–dominated vs fast-mode–dominated classification on the independent test set using the 1D-CNN with attention; the optimal threshold is marked using the Youden J statistic, and AUC with 95% CI. (E) Cluster-stratified ROC curves for flexible vs non-flexible classification, evaluated separately within each GI cluster on the test set. (F) Cluster-stratified ROC curves for slow-mode vs fast-mode dominance classification, evaluated separately within each GI cluster on the test set. (G) Normalized confusion matrix for flexible vs non-flexible classification on the test set at a probability threshold of 0.5 (values shown as proportions). (H) Confusion matrix for slow-mode vs fast-mode dominance classification on the test set at a probability threshold of 0.5 (values shown as proportions). (I) ROC comparison for flexible vs non-flexible classification across model classes trained on GI descriptors evaluated on the same test set. (J) Residue-wise slow- and fast-mode RMSF contributions for 4ALZ (chain A), a high slow-fraction exemplar. (K) Residue-wise slow- and fast-mode RMSF contributions for 2ERL (chain A), a low slow-fraction exemplar. (L) ANM displacement vectors for 4ALZ (chain A) illustrating a coherent, collective deformation. (M) ANM displacement vectors for 2ERL (chain A; mating pheromone Er-1) illustrating more localized and heterogeneous motion.

Across GI-defined structural clusters, both labels were approximately balanced in the test set (Fig. 3A and B), indicating that neither task is trivially driven by cluster membership alone. For the global flexibility task, the model achieved good discrimination on the full test set (AUC = 0.77; Fig. 3C), while the slow/fast dominance task was more separable (AUC = 0.91; Fig. 3D), supporting the idea that GI descriptors are global topology features which align with mode composition more than amplitude. When stratifying by GI cluster, ROC curves remained well above chance for both tasks (Fig. 3E and F), with weaker performance in cluster 1 likely reflecting its small test-set size (*n = *19) and greater heterogeneity, possibly due to the scarcity of homologs and reduced fold-level coherence as seen in Fig. 2C–E.

At a fixed decision threshold (0.5), confusion matrices showed 75.0% correct identification of non-flexible proteins and 69.1% correct identification of flexible proteins (Fig. 3G), and 76.9%/87.9% correct classification for fast-/slow-dominated proteins, respectively (Fig. 3H). Finally, compared against alternative GI-based models and Backflip, the CNN with attention yielded the highest ROC performance among GI-based approaches, whereas Backflip achieved the strongest overall classification performance on the test set (Fig. 3I).

To illustrate the biological meaning of the slow-fraction classifier, we examined representative proteins from opposite ends of the spectrum together with residue-wise slow/fast RMSF profiles. The high slow-fraction exemplar 4ALZ (chain A) is YscD, a basal-body component of the Yersinia injectisome whose function depends on structural elasticity within the type III secretion system (Kudryashev *et al.* 2013). Its dynamics are dominated by slow-mode contributions and coherent ANM displacements (Fig. 3J and L), consistent with prior evidence that YscD undergoes hinge-like interdomain rearrangements and contributes to basal-body elongation under membrane stress. In contrast, the low slow-fraction exemplar 2erlA is the mating pheromone Er-1, a small signaling protein whose high-resolution structure showed little overall rigid-body motion but substantial side-chain motion relative to the backbone as mentioned by Anderson *et al*. (1996). In line with that behavior, 2erlA was predicted to have relatively stronger fast-mode contributions and a less globally coordinated displacement pattern (Fig. 3K and M), supporting its interpretation as a fast-enriched example. Animations illustrating these motions are provided in the Supplementary Materials.

For both classification tasks, training and validation loss decreased rapidly during early epochs and then plateaued, indicating stable convergence under five-fold cross-validation. The flexibility task showed a modest late train–validation divergence with mild overfitting, whereas the slow/fast dominance task maintained closer alignment between curves with a more separable label signal (Fig. S1A–B, available as supplementary data at *Bioinformatics* online).

Targeted ablation and attribution analyses converged on a sparse subset of GI positions as consistently high-importance features across both classifiers (Fig. S1C–D, available as supplementary data at *Bioinformatics* online). Cross-method agreement spanning univariate associations, single-feature baselines, permutation importance, and attention-based contrasts argues that most predictive signal is concentrated in a limited set of low- and mid-order GI components (1 through 15). This concentration suggests that these descriptors capture global backbone geometry, such as overall entanglement/handedness (e.g. writhe/average crossing) and longer-range curvature correlations, rather than uniformly distributed local effects.

### 3.3. Deep learning on GI descriptors predicts mean and slow-mode RMSF

**Figure 4:**
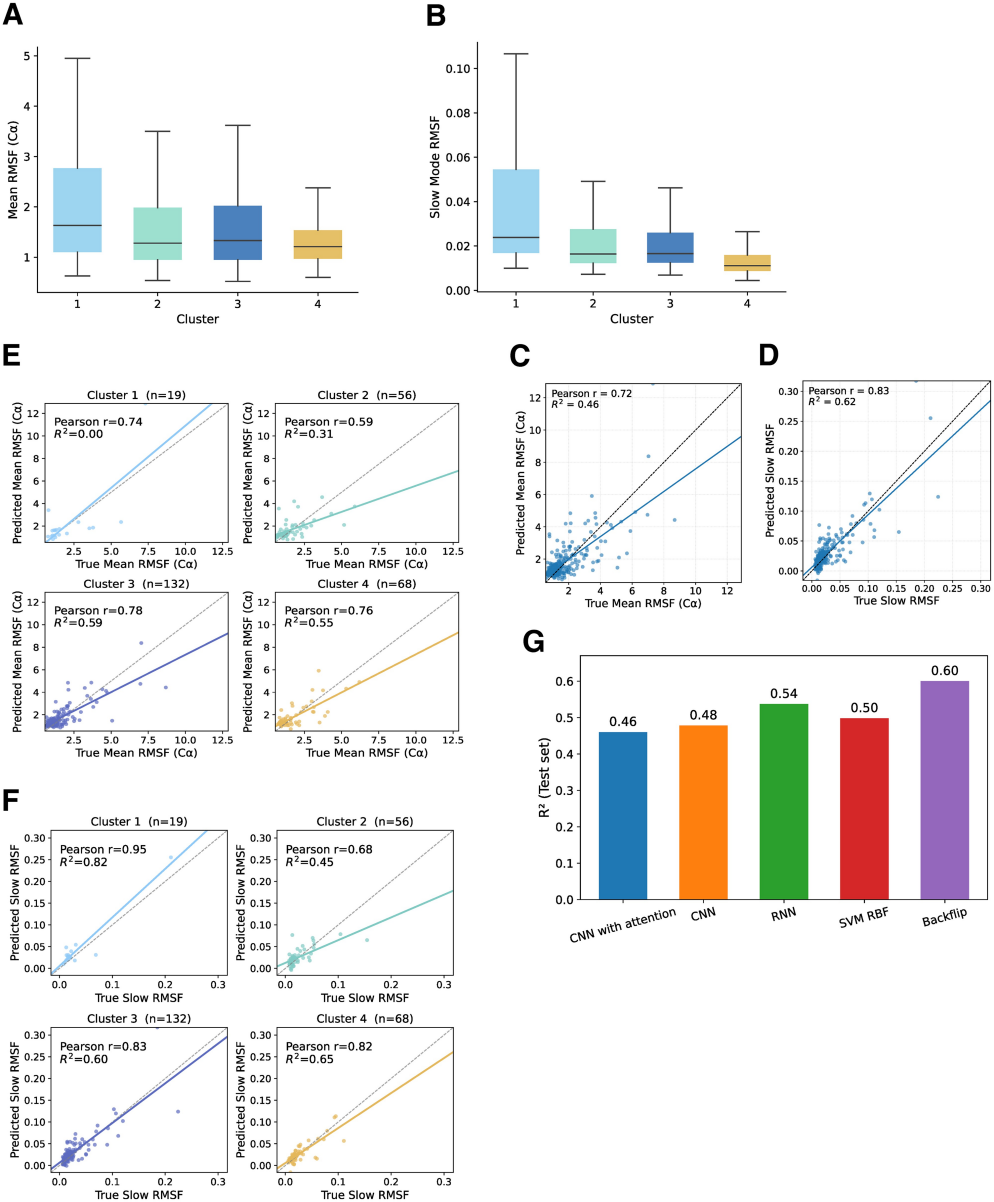
Deep learning on GI descriptors predicts mean and slow-mode RMSF. (A) Distribution of mean per-protein Cα RMSF across GI clusters for the test set, shown as per-cluster boxplots. (B) Distribution of GNM-derived slow-mode RMSF across GI clusters for the test set, shown as per-cluster boxplots. (C) Regression performance for mean RMSF on the test set predicted versus true per-protein values with Pearson *r* and *R*^2^ reported in-panel; dashed line indicates the identity ($\hat{y}=y$) and the solid line shows the ordinary least squares fit. (D) Regression performance for slow-mode RMSF on the test set: predicted versus true per-protein values with Pearson *r* and *R*^2^ reported in-panel; dashed line indicates the identity ($\hat{y}=y$) and the solid line shows the ordinary least squares fit. (E) Cluster-stratified regression for mean RMSF, showing predicted versus true values within each GI cluster on the test set; per-cluster Pearson *r* and *R*^2^ reported in-panel). (F) Cluster-stratified regression for slow mode RMSF, showing predicted versus true values within each GI cluster on the test set; per-cluster Pearson *r* and *R*^2^ reported in-panel). (G) Model comparison for mean RMSF regression, reporting test-set *R*^2^ across architectures trained on GI descriptors.

In addition to binary classification, we evaluated GI descriptors in a regression setting by training the same 1D-CNN with attention to predict continuous per-protein fluctuation amplitudes on the held-out 275-protein test set. We considered two targets: the mean Cα RMSF from ATLAS MD trajectories and the GNM-derived slow-mode RMSF computed from low-frequency mode subsets (Fig. 4A–F).

Across GI clusters, mean RMSF and slow-mode RMSF exhibited broadly similar distributional trends, with cluster 1 showing a small number of high outliers and cluster 4 displaying a comparatively narrow range (Fig. 4A–B). On the test set, predicted mean RMSF correlated with the ground truth (Pearson *r = *0.72; *R*^2^ = 0.46; Fig. 4C), whereas slow-mode RMSF was predicted more accurately (Pearson *r = *0.83; *R*^2^ = 0.62; Fig. 4D), consistent with the idea that GI descriptors capture global topology that aligns more strongly with collective-mode as seen in Fig. 3D. Cluster-stratified regression for mean RMSF showed strong performance in clusters 3–4 but remained weaker in cluster 1 (*n = *19; Fig. 4E), likely reflecting both limited sample size and a smaller dynamic range within predominantly α-helical folds, which can depress *R*^2^ even when rank correlation appears moderate. By contrast, performance within each cluster was consistently higher for slow mode RMSF across all clusters (Fig. 4F). Model comparisons on the mean-RMSF regression task showed that an RNN achieved the highest test-set *R*^2^ among GI-based models (0.537), with CNN-based models and SVM-RBF performing similarly but slightly lower, whereas Backflip achieved again the highest overall performance (Fig. 4G). Backflip also required less inference time on the same test set, completing prediction in 9 s compared with 35 s for our 1D-CNN. Although our method does not achieve state-of-the-art RMSF prediction performance, GIs remain a compact and interpretable alternative for structure-based inference of global protein dynamics.

Training curves indicated stable convergence for both regression tasks (Fig. S2A–B, available as supplementary data at *Bioinformatics* online), and targeted ablation/attribution heatmaps suggest that regression performance draws on a broader set of GI positions than classification, implying that continuous targets depend on multiple complementary geometric/topological cues (Fig. S2C–D, available as supplementary data at *Bioinformatics* online).

### 3.4. Coarse-grained multi-label function prediction from GI topology features

We additionally explored whether GI descriptors could support protein function prediction using Gene Ontology (GO)-based labels, but encountered substantial annotation sparsity and label heterogeneity across the ATLAS set. To mitigate this, we collapsed annotations to a coarse-grained set of functional categories, filtered rare labels with insufficient support, and treated proteins as multi-label instances, evaluating performance by comparing predicted label sets against curated label sets (Fig. S1E–F, available as supplementary data at *Bioinformatics* online). On the independent test set, the model achieved a micro-average precision–recall AUPRC of 0.28, substantially above the baseline prevalence (0.07), with class-wise average precision varying strongly with label support and functional category (Fig. S1F, available as supplementary data at *Bioinformatics* online).

## 4 Discussion

This study shows that a compact representation of protein topology (a 30 GI descriptor) computed from the Cα trace captures conserved structural organization across a diverse set of proteins. GI-based PCA partitioned ATLAS into four clusters that differed in secondary structure composition and, importantly, recapitulated established biological relationships: homologous protein pairs overwhelmingly mapped to the same GI cluster, and ECOD fold families showed strong cluster-specific enrichment. These results tell us that GIs do not merely compress geometry, but encode signatures at the fold level that are conserved across evolution and reflected in domain architecture.

Building on this structural stratification, we found that GI descriptors support accurate prediction of dynamic properties at two complementary resolutions. First, GIs fed into our 1D-CNN enabled classification of global flexibility (mean RMSF above/below the dataset mean) with good test-set discrimination, indicating that the magnitude of conformational fluctuation is partially constrained by the overall backbone topology. Second, our model achieved an even better performance when evaluating slow/fast dominated proteins, potentially explained by GIs reflecting collective deformations. Biologically, this aligns with the intuition that fold architecture encodes “preferred directions” of collective deformation, such as hinge-like bending, twisting, and domain rearrangements, whereas absolute RMSF can be amplified or dampened by local elements (loops, termini, surface exposure) and sequence-specific interactions that are not explicitly represented by GIs.

Regression analyses reinforced the same hierarchy of predictability. Mean RMSF could be predicted with moderate accuracy, whereas slow-mode RMSF was predicted more strongly, indicating a tighter relationship between fold topology and collective motion. This distinction matters biologically because slow collective modes often underlie functional conformational changes (domain closure, allosteric transitions, coordinated rearrangements), while faster components primarily reflect local fluctuations.

Cluster-stratified analyses indicate that structural context influences predictability. Cluster 1 contained the fewest proteins across train/validation/test, was predominantly α-helical, and showed the weakest biological coherence by both homology pairing and ECOD fold-family coverage. These factors likely limit the amount of learnable signal available for flexibility and mean-RMSF prediction within this cluster. Notably, performance degraded far less overall for slow-mode objectives, highlighting that collective motions are more directly constrained by fold geometry captured by GI descriptors than total fluctuation amplitude.

The observation that different model classes performed best for different objectives (an attention based CNN for classification and an RNN for regression) further indicates that information about dynamics is distributed across GI features in a task dependent manner. Targeted ablation analyses reinforce this interpretation: categorical decisions can be driven by a small subset of GI positions, whereas continuous amplitude prediction benefits from integrating broader patterns across the full GI vector.

Finally, our exploratory attempt to predict broad function labels from GI descriptors revealed both promise and current limitations. Performance exceeded baseline prevalence, indicating that topology at the fold level contains some relevant signal, consistent with the well-known coupling between domain architecture and molecular role. However, incomplete annotation, heterogeneous label granularity, and multifunctionality impose a ceiling on what can be learned from topology alone; many functional determinants are driven by sequence and chemistry (active-site residues, binding motifs, PTM sites) and may not be recoverable from a purely geometric encoding.

Overall, GI descriptors provide a scalable bridge between protein architecture and dynamics, enabling rapid prediction of flexibility and collective-motion bias across diverse folds and motivating sequence-augmented models for improved functional inference.

## Supplementary Material

btag446_Supplementary_Data

## Data Availability

The ATLAS molecular dynamics trajectories and associated annotations analyzed in this study are publicly available through the ATLAS database (https://www.dsimb.inserm.fr/ATLAS). All code used for Gaussian-integral calculation, model training, evaluation, and figure generation, together with the analysis-ready input files required to reproduce the results, is publicly available at https://github.com/fvilicich/gaussian_integral.
