# Supplementary material for: Inferring dynamic information from protein structures by Gaussian integrals and deep learning
Source: Bioinformatics. 2026 Jun 24;42(7):btag446. doi: 10.1093/bioinformatics/btag446 (PMC13371761; doi:10.1093/bioinformatics/btag446)
Supplement: btag446_Supplementary_Data [file btag446_supplementary_data.zip › Supplementa_Materials.pdf]

Inferring Dynamic Information from Protein Structures by Gaussian Integrals and Deep Learning

Supplemental Materials

Felipe Vilicich[a] , Nicolas Bottino[d], Zhaoqian Su[b], Shanye Yin[c], Yinghao Wu[a] *

[a] Department of Systems and Computational Biology, Albert Einstein College of Medicine,
1300 Morris Park Avenue, Bronx, NY, 10461.
[b] Data Science Institute, Vanderbilt University, 1001 19th Ave S, Nashville, TN, 37212.
[c] Department of Pathology, Albert Einstein College of Medicine, 1300 Morris Park Avenue,
Bronx, NY, 10461.
[d] Departamento de Ciencias Aplicadas, Universidad Siglo 21, De los Latinos 8555, X5147
Córdoba, Argentina

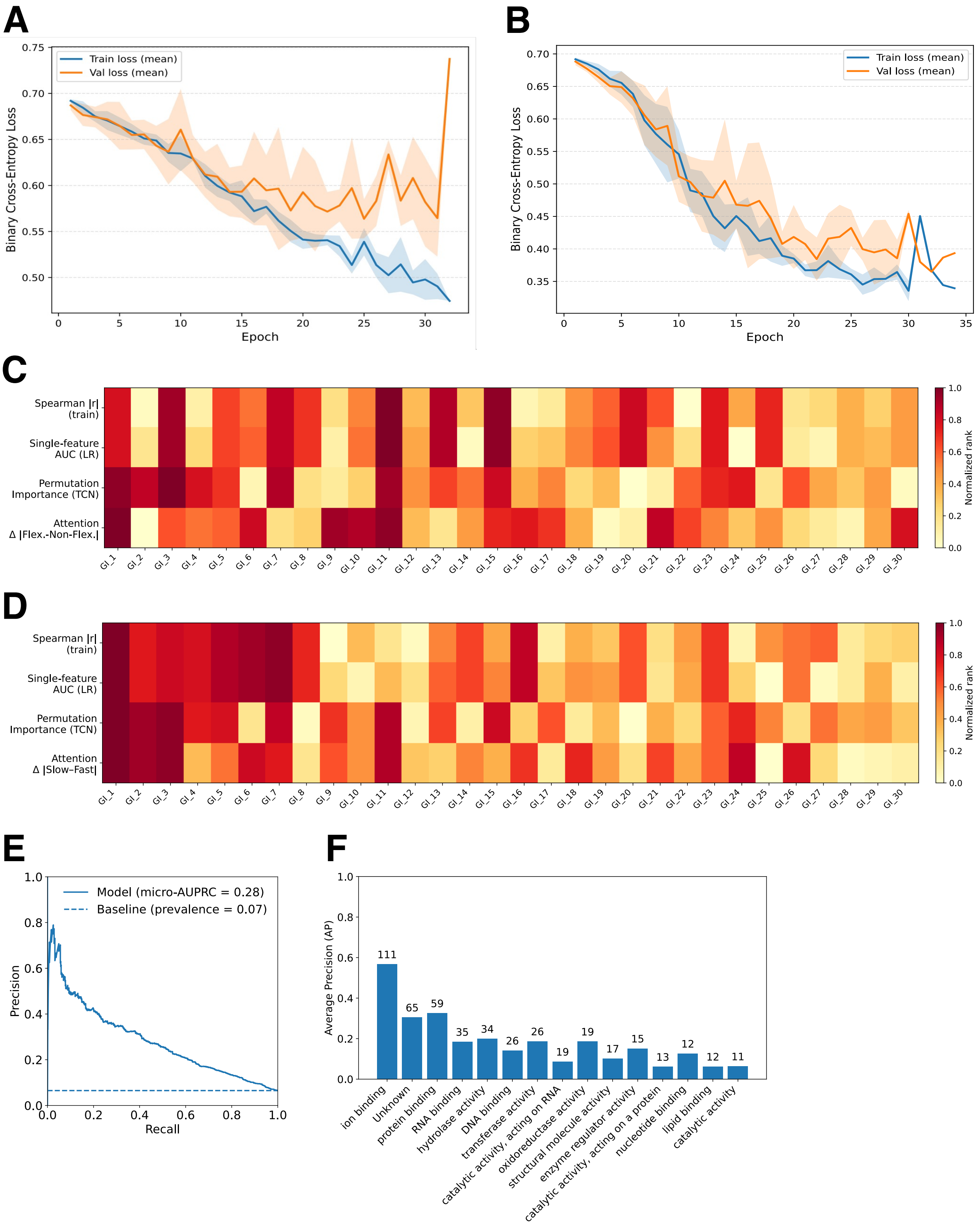

Supplementary Figure 1. Training dynamics, interpretability, and exploratory function prediction. A) Training and validation loss curves (mean ± 1 SD across folds) for the flexible vs non-flexible classification task. **B)** Training and validation loss curves (mean ± 1 SD across folds) for the slow-mode–dominated vs fast-mode–dominated classification task. **C)** Targeted ablation/attribution heatmap for flexible vs non-flexible classification, summarizing GI feature importance across four methods (Spearman |r| on training data, single-feature AUC using logistic regression, permutation importance on the CNN with attention, and attention-difference between classes), shown as normalized ranks across GI positions. **D)** Targeted ablation/attribution heatmap for slow vs fast dominance classification, summarizing GI feature importance across the same four methods as in **C)**, shown as normalized ranks across GI positions. **E)** Micro-averaged precision–recall curve for the coarse-grained multi-label GO function prediction task on the independent test set, with the baseline precision equal to label prevalence. **F)** Per-class AP for the function prediction task on the independent test set; numbers above bars indicate the number of test proteins (support) annotated with each GO functional category.

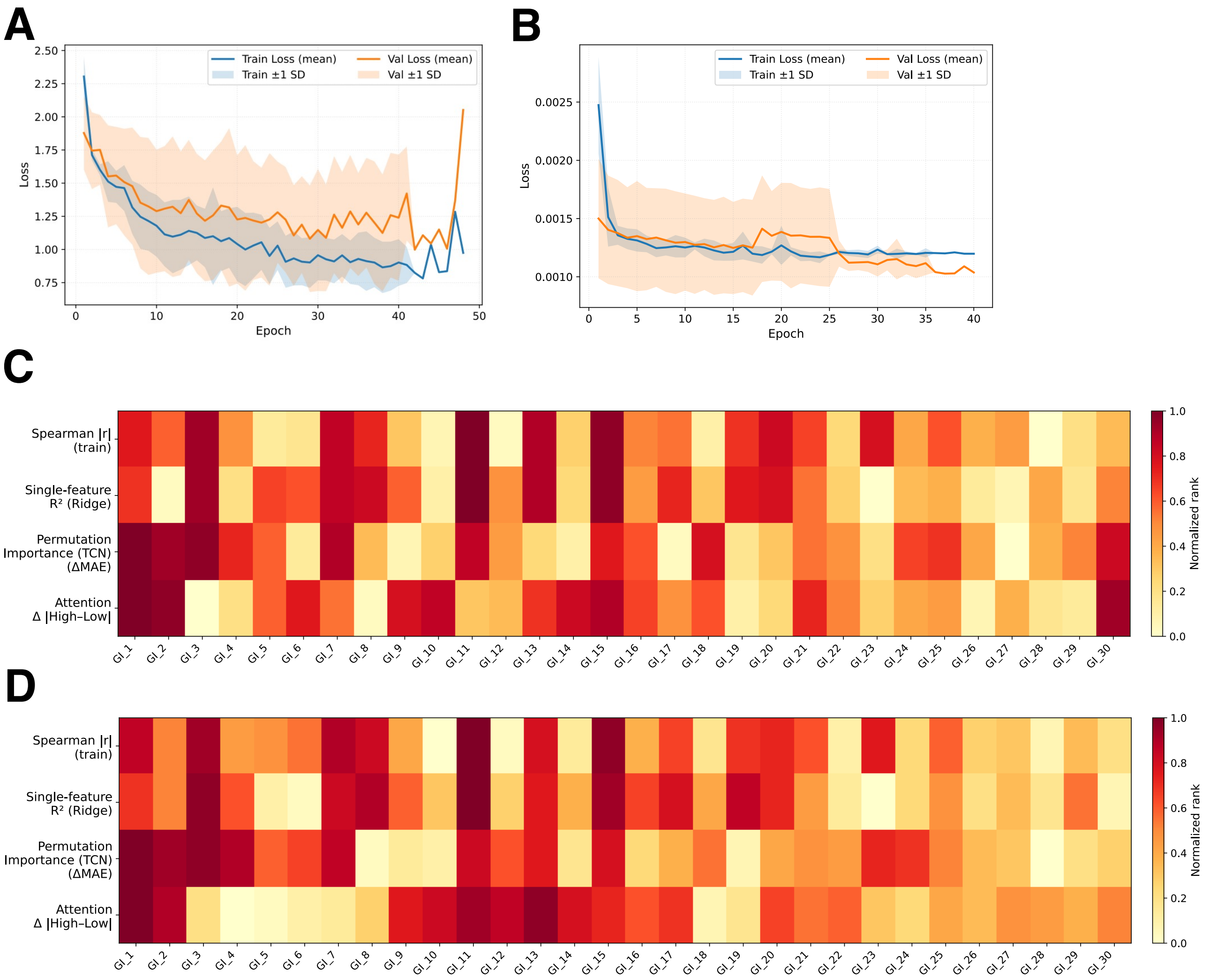

Supplementary Figure 2. Training dynamics and targeted ablation for regression tasks. A) Training and validation loss curves (mean ± 1 SD across folds) for **mean** RMSF regression. **B)** Training and validation loss curves (mean ± 1 SD across folds) for slow-mode RMSF regression. **C)** Targeted ablation/attribution heatmap for mean RMSF regression, summarizing GI feature importance across four methods (Spearman |r| on training data, single-feature performance using linear regression (R^2), permutation importance on the CNN with attention (ΔMAE), and attention-based differences), shown as normalized ranks across GI positions. **D)** Targeted ablation/attribution heatmap for slow-mode RMSF regression, summarizing GI feature importance across the same four methods as in **C)**, shown as normalized ranks across GI positions.
